# Important factors for public acceptance of the final disposal of contaminated soil and wastes resulting from the Fukushima Daiichi nuclear power station accident

**DOI:** 10.1371/journal.pone.0269702

**Published:** 2022-06-22

**Authors:** Momo Takada, Kosuke Shirai, Michio Murakami, Susumu Ohnuma, Jun Nakatani, Kazuo Yamada, Masahiro Osako, Tetsuo Yasutaka

**Affiliations:** 1 Institute for Geo-Resources and Environment, National Institute of Advanced Industrial Science and Technology, Tsukuba, Ibaraki, Japan; 2 Social Safety and Industrial Innovation Division, Mitsubishi Research Institute, Inc., Chiyoda-ku, Tokyo, Japan; 3 Department of Health Risk Communication, Fukushima Medical University School of Medicine, Fukushima City, Fukushima, Japan; 4 Department of Behavioral Science, Graduate School of Letters, Hokkaido University, Sapporo, Japan; 5 Department of Urban Engineering, The University of Tokyo, Bunkyo-ku, Tokyo, Japan; 6 Fukushima Regional Collaborative Research Center, National Institute for Environmental Studies, Miharu, Fukushima, Japan; 7 Material Cycles Division, National Institute for Environmental Studies, Tsukuba, Ibaraki, Japan; Texas A&M University System, QATAR

## Abstract

Large-scale decontamination work has been carried out in the aftermath of the Fukushima Daiichi nuclear power station accident in Japan in 2011. The soil that was removed and the wastes that were generated during the decontamination will be finally disposed of outside Fukushima Prefecture by 2045. To ensure successful and socially acceptable implementation of this final disposal process, it is essential to have a good understanding of what is considered important by the public. We used a choice-based conjoint analysis in the form of a web-based questionnaire to examine the relative importance of several factors in the choice of the final disposal sites of the removed soil and incinerated ash of the wastes. The questionnaires covered four attributes and 12 levels, namely the distance between the disposal site and a person’s residential area, procedural fairness (decision process), distributive fairness (direct mitigation of inequity through multiple siting locations), and the volume and radioactivity of the substances to be disposed. Responses were received from 4000 people nationwide, excluding Fukushima residents. The results showed that the respondents gave high importance to choosing sites that were far from residential areas and to the two types of fairness, especially distributive fairness. The respondents showed no preference for the volume and radioactivity. This indicates that the public cares about the fairness of the siting for the final disposal sites and feels uncomfortable with plans for a final disposal site located close to them. Distributive fairness is necessary to pursue consensus in addition to procedural fairness.

## Introduction

The Great East Japan Earthquake of 2011 caused an accident at the Fukushima Daiichi nuclear power station. The event was rated as a Level 7 severe accident by the International Nuclear Event Scale, which is equal to the level of the accident at Chernobyl in 1986 [1]. In the period between the accident and March 2018, the national and prefectural governments carried out decontamination work in 43 of the 59 local municipalities of Fukushima Prefecture, the location near the nuclear power station and the most heavily contaminated area. The decontamination work, which included residential areas, farmland, forests, and roads close to living areas and excluded the difficult-to-return zones (approximately 2.4% of the prefecture’s area) [2], generated approximately 14 million m^3^ of decontaminated soil and organic matter [3]. Of this waste, 80% is estimated to have radiocesium activities less than 8000 Bq/kg [4]. By the end of March 2022, the decontaminated soil and the organic matter will have been transported to an interim storage facility that has an area of approximately 1600 ha over the two municipalities where the Fukushima Daiichi nuclear power station is located, and the soil and incinerated ash of the organic matter will be stored separately. In line with the Japan Environmental Storage & Safety Corporation Law (Law No. 44 of 2003, promulgated on May 16, 2003), the Japanese government has decided that the removed soil and incinerated ash will be transported out of Fukushima Prefecture for final disposal by 2045, and the removed soil will be recycled and used as civil engineering materials throughout Japan.

The importance of the non-technical aspects of the disposal and management of radioactive wastes has been emphasized worldwide. The OECD/NEA reported in 2020 that the social aspect was very important in optimizing radioactive waste management and needed to be based on social involvement [5]. The final disposal of the contaminated soil and wastes outside Fukushima Prefecture presents social issues. Indeed, the Japanese government recognizes that, for the disposal plan to be accepted by the public, social aspects, such as public understanding, must be considered along with the outcomes of research and development about the reduction in the volume of the removed soil and the incinerated ash [3].

This initiative marks the first final disposal worldwide of contaminated soil and waste gathered during decontamination following a nuclear power plant accident, and it has notable features, including the social perspectives, that mean that it differs from traditional waste disposal (Table 1). The contaminated soil and waste that result from a radioactive accident seem extremely negative, and will be considered very differently by people than wastes conventionally derived from people’s lives and normal power generation. The contamination or hazardous level and management period of the materials also differ. The decontaminated soil and waste in this study need to be managed more strictly than municipal solid waste, but not as strictly as high-level radioactive waste. That the waste should be finally disposed of outside Fukushima Prefecture has been decided legally, and the original area where the soil and waste were generated has been excluded from the process for selecting candidate disposal sites. The candidate sites are therefore located throughout Japan and exclude Fukushima, and the residents close to the candidate sites are all Japanese and exclude the residents of Fukushima Prefecture. Potential disposal sites for high-level radioactive wastes that require geological disposal are subject to geological restrictions; there are no geological restrictions on the potential disposal sites for the removed soil and the incinerated ash from Fukushima, so these sites are distributed all over Japan.

**Table 1:** Social perspectives on the disposal of traditional wastes and the contaminated soil and waste from the Fukushima accident.

	Municipal solid waste	High-level radioactive waste	Decontaminated soil and waste (this study)
**Source**	People’s daily lives	Nuclear power generation	Accident due to nuclear power generation
**Hazardous level**	The level of contamination is manageable in a typical waste disposal site	The level of contamination that requires geological disposal and tens of thousands of years of management	The level of contamination is manageable in a typical waste disposal site, and the management period is several hundred years
**Disposal site**	Within the area of waste generation	Selection from across a country, including a region of the waste generation^a^	Selection from across a Japan except Fukushima Prefecture, under law^b^

^a^ Assuming disposal in own country

^b^ Japan Environmental Storage & Safety Corporation Law (Law No.44 of 2003, Promulgated on May 16, 2003)

The final disposal of contaminated soil and wastes generated by the Fukushima accident can be characterized as above. It is a type of NIMBY (not in my backyard) facility, a term coined for disposal sites of municipal, industrial, and radioactive wastes, and nuclear facilities. Many studies have been conducted on the public acceptance of facility siting. Studies of the management and disposal facilities for non-radioactive wastes have shown that resistance to the facility increases with decreasing distance between the proposed facility and a residential area [6–9]. Similarly, the degree of acceptance decreases with increasing perception of the risk of a high-level radioactive waste facility, and increases with increasing trust in the government and its agencies [10, 11]. The existence of a radioactive waste disposal site is believed to damage the image of a region, which thus has a negative impact on acceptance [12, 13].

Procedural fairness also influences public acceptance. A review of the process used for siting hazardous waste management facilities in four Canadian provinces in the 1980s and 1990s found that access to public decision-making, or procedural fairness, not top-down decision-making, was important for successful siting [14]. A telephone survey conducted in the United States in 2008–2009 showed that acceptance of a decision to build a nuclear facility was positively influenced by perception of the fairness of the decision-making process, or procedural fairness [15]. Questionnaire surveys in Japan in 2011 and 2012 also showed that the perception of procedural fairness had a positive impact on public acceptance of the geological disposal of high-level radioactive waste [13].

Fairness also includes distributive fairness, which focuses on the distributive aspects of the outcomes of social actions and decisions [15]. Monetary compensation is commonly used to correct unfairness in the allocation of burdens, such as the locations of waste disposal sites. However, several studies have shown that attempts to maintain equilibrium through monetary compensation do not always lead to public acceptance [16–18]. For example, Yokoyama et al. [19] found that direct inequity mitigation, i.e., actual burden sharing, affects public acceptance. A web-based questionnaire survey throughout Japan to gather information about the acceptance of recycling the decontaminated soil from the Fukushima accident as civil engineering materials showed that public acceptance was higher for use in multiple locations than in a single location [19].

The factors that affect public acceptance may also be important for the final disposal of contaminated soil and waste outside Fukushima Prefecture. However, the relative importance of these factors is unique to each case, and should also be unique to the final disposal. Furthermore, the importance may change with time as the final disposal plan proceeds. Although individuals may consider different factors important, the implementation of the final disposal may be more socially acceptable if the factors that are important to the public are clarified.

In this study, the relative importance of these factors over a period of 25 years, until the completion of the final disposal, was examined using a web-based choice-based conjoint analysis. We attempted to determine (1) the major factors that influenced public acceptance about the final disposal of the contaminated soil and wastes outside Fukushima Prefecture and their relative importance, and (2) whether the important factors varied between different groups of people.

## Materials and methods

### Conjoint analysis

The conjoint analysis method used in this study was developed in psychometrics in the 1960s [20]. The method is designed to evaluate people’s conscious or unconscious preferences for a product or service containing multiple elements, and allows the overall evaluation of an object to be divided into individual attributes, which are given partial utility values that reflect their importance. This method is often used in marketing, as well as other fields including environmental assessment [21]. Several studies have reported how public preferences have been incorporated into the selection process for radioactive or non-radioactive waste disposal sites to improve the acceptability of the decision making [22–24].

In conjoint analysis, the evaluation target is represented by a profile composed of various attributes and attribute levels, and this profile is evaluated by questionnaire respondents. In this example, the attributes might be the distance to the disposal site and the decision-making process. The attribute levels for distance could be neighborhood, municipality, or prefecture, and the attribute levels for the decision-making process could be top-down, offering comments, or a reflection of opinions. In conjoint analysis, the total of the partial utilities of all the attributes represents the total utility [22–24].

### Samples

The respondents in this study were people aged between 20 and 69 years old who live in 46 prefectures throughout Japan, except Fukushima Prefecture. This sample group was chosen because the final disposal sites could be in any area of Japan apart from Fukushima Prefecture, and people in this age range are the main stakeholders in the consensus building. A web-based questionnaire survey carried out in January 2021 generated a total of 4000 responses. The questionnaire comprised two different surveys, one for removed soil and one for incinerated ash. There were 2000 responses for each of these surveys (each survey was continued until 2000 responses were obtained), and the respondent groups differed between the two surveys. The collected data were evenly distributed by geographical area (across eight regional blocks in Japan, namely Hokkaido, Tohoku (excluding Fukushima Prefecture), Kanto, Chubu, Kinki, Chugoku, Shikoku, and Kyushu region (including Okinawa), gender, and age group (20s, 30s, 40s, 50s, and 60s). Respondents to the survey were incentivized with web points, which could be substituted for cash, and were worth approximately 20 Japanese Yen (0.17 US$). Respondents first entered basic information (e.g., age, gender, prefecture, children), and then had to watch a preliminary explanation about the final disposal for at least 30 seconds before beginning to answer. The material included an overview of the environmental contamination caused by the accident, the decontamination and the interim storage facility, the plan for final disposal outside Fukushima Prefecture to be completed in 30 years, radiation protection and half-lives of ^137^Cs and ^134^Cs, and volume reduction technology. The contents about the environmental safety of the final disposal are shown in S1 Fig. It was made clear to the respondents that, regardless of whether volume reduction technology is applied, the final disposal site would be safe for humans and the environment. This supports the understanding of the attributes and attribute levels of the conjoint questionnaire.

The present study was approved by the research ethics committee of Hokkaido University (receipt number, FY2020-08 (removed soil), FY2020-16 (incinerated ash)). All respondents participated voluntarily and gave consent for their responses to be used for research purposes and published. Respondents were first given detailed information about their participation via text, including a brief overview of the survey and the intended use of the data, and were also explicitly informed that the submission of their answer to the questionnaire will constitute the participant providing consent to participate.

### Questionnaire design

We chose four attributes for our study, three of which were based on previous studies, namely the distance from the residential area to the final disposal site [6–8], and two types of fairness, procedural fairness, which was related to the decision process [13–15], and distributive fairness, related to the total number of final disposal sites [19]. The fourth attribute was the volume and radioactivity concentration of the removed soil and incinerated ash and was related to the research and development on volume reduction technology, carried out by the Ministry of the Environment and used to foster public understanding [3]. There is a need to examine the degree to which the public accepts the volume reduction. Each of the four attributes have three attribute levels (Table 2).

- *Distance from the residential area to the final disposal site*. This is to examine the respondents’ perceptions of having final disposal sites close to them. Respondents were asked their preference for siting in their neighborhood, their municipality, or their prefecture.
- *Decision process*. This examined the preference for procedural fairness. There were three levels, according to the degree of influence of the public on the decision-making, namely top-down decision-making by a mayor/local governor, decision-making by a mayor/local governor after calling for residents’ comments, and decision-making by a mayor/local governor after discourse with residents.
- *Total number of final disposal sites*. This examined the preference for distributive fairness. We followed the direct inequity mitigation approach used by Yokoyama et al. [19] to evaluate the locations of the disposal sites. We used the following categories: only one location in Japan; a total of eight locations, meaning one location in each of the eight regional divisions, or a total of 46 locations, meaning one in each prefecture in Japan, excluding Fukushima Prefecture.
- *Volume and radioactivity concentration*. This examined the preference for volume reduction technology. Without the volume reduction, the radioactivity is relatively low, but the disposal site needs to cover a large area because of the large volume. In contrast, the volume reduction technology reduces the volume but increases the radioactivity, meaning the disposal area can be smaller but the management is more complicated and residents may not be so accepting (S1 Fig).

**Table 2:** Conjoint attributes and attribute levels.

Attribute	Attribute level
**Location of final disposal site**	Neighborhood
	Within your municipality
	Within your prefecture
**Decision process**	Top-down. Mayor/local governor decide to accept final disposal site.
	Offering comments. Comments from residents are called for, and the mayor/local governor decide to accept final disposal site.
	Reflection of opinions. Discourse by residents is held, and a chief decides to accept final disposal site.
**Total number of final disposal sites**	Only one
	Eight in total, one in each region
	46, one in each prefecture except Fukushima Prefecture
**Volume and radioactivity** *	Medium radioactivity in large quantity (No treatment)
	Removed soil, ca. 13 million t; 8000 Bq/kg
	Incinerated ash, ca. 460,000 t; 33,000 Bq/kg
	High radioactivity in medium quantity (Volume reduction treatment)
	Removed soil, 1.3 million t; 76,000 Bq/kg
	Incinerated ash, 1500 t; 11.4 million Bq/kg
	Very high radioactivity in small quantity (Super volume reduction treatment)
	Removed soil, 4000 t; 23 million Bq/kg
	Incinerated ash, 8 t; 1980 million Bq/kg

* Respondents to the surveys about the removed soil and incinerated ash were asked about their preferences for different volume and radioactivity levels.

**Fig 1:**
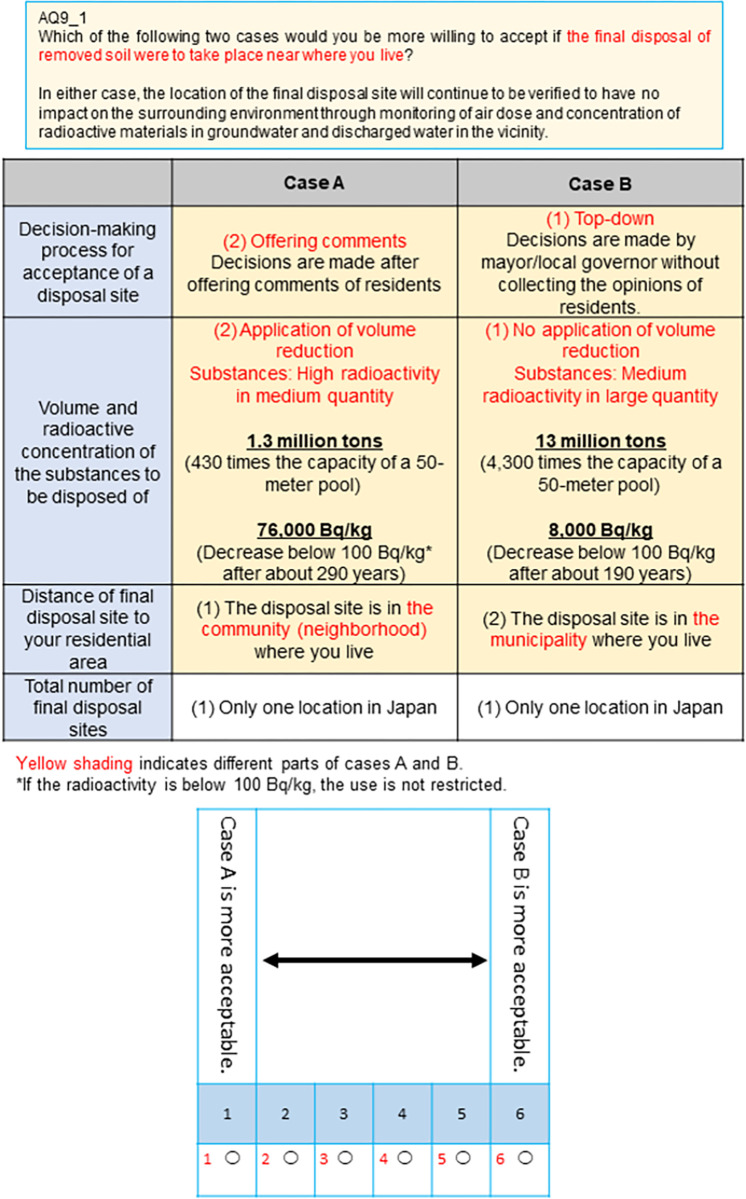
An example of a question used in the choice-based conjoint analysis questionnaire. Two final disposal site scenarios (profiles) were presented in each selection screen. The levels of the attributes were different in each selection screen. The questionnaire was in Japanese, and was translated into English for this manuscript.

There were three attribute levels for each of the four attributes, and there were 81 proposed final disposal siting conditions (hereinafter called profiles). We had too many (81) profiles to use in the questionnaire, thus we used an orthogonal design (IBM SPSS Statistics version 27) to narrow down the number of profiles to nine so that all of the criteria were equally represented. Two of each profile were presented in pairs, and respondents gave an answer on a scale of 1 to 6 about which they preferred (Fig 1). Pairing the nine profiles gave 36 pairs. We then excluded pairs where one profile was clearly more acceptable, which left 25 pairs. Each of the 2000 respondents were divided into five groups with 400 respondents in each, and each group responded to five pairs of profiles. These five pairs were shown to each respondent in a random order, such that the respondents viewed them in a different order.

As well as selecting a more acceptable profile, respondents were asked to answer a question on their basic knowledge of the final disposal using a four-point scale, and to answer 15 questions about their perception of the risk of health effects from radioactive materials and four questions about their trust in the Ministry of the Environment, with answers on a five-point scale (Table 3). The risk perception questionnaire was based on that of Slovic [25], and the trust questionnaire was based on those of Visschers & Siegrist [26] and Nakayauchi & Cvetkovich [27]. The questionnaire and the reliability coefficients (Cronbach’s alpha) for the risk perception and trust are shown in Table 3. The risk perception questions included dread factors (2–11 in Table 3) and unknown factors (12–16 in Table 3), so the total score was used in the analysis as a category of risk perception, because Cronbach’s alpha had values of 0.93 and 0.92 for the removed soil and the incinerated ash, respectively. The respondents were grouped according to their scores for each of the three question categories, and the importance of each attribute was examined. The frequency distributions of the total scores of the risk perception and trust were mound-shaped distributions with no other notable peaks. The total scores for ‘undecided’ were 45 for risk perception and 12 for trust, which were extremely high and accounted for approximately 17% for risk perception and approximately 30% for trust. Therefore, the respondents were divided into three groups, namely for a total score for ‘undecided,’ and scores higher, and lower, than that.

**Table 3:** Questionnaire items for grouping respondents and the reliability coefficients.

Question	Cronbach’s alpha	
	Removed soil	Incinerated ash
**Basic knowledge on final disposal**		
1. Do you know that the law stipulates that the removed soil/incinerated ash is disposed of outside Fukushima Prefecture within 30 years after the start of interim storage?	-	-
**Risk perception of health effects of radioactive materials**		
If a final disposal site for removed soil/ incinerated ash is built in your municipality, what do you think of the following?	0.93	0.92
2. Final disposal is intuitively frightening.		
3. Final disposal site causes unnoticed damage.		
4. Final disposal site nearby causes massive damage.		
5. Final disposal site causes many victims at once.		
6. Final disposal site causes life-threatening damage.		
7. Damage caused by a final disposal site varies from person to person.		
8. Damage caused by a final disposal site affects future generations.		
9. Damage caused by final disposal site continues to increase.		
10. Reducing damage caused by a final disposal site is difficult.		
11. I can avoid a damage caused by a final disposal site by myself.*		
12. Damage caused by a final disposal site is not immediately apparent.		
13. Process of damage caused by a final disposal site is not well understood.		
14. Damage caused by a final disposal site is unpredictable.		
15. Damage caused by a final disposal site has never been seen before.		
16. Damage caused by a final disposal site is not scientifically understood.		
**Trust in the Ministry of the Environment**		
17. Trust as the entity for final disposal	0.95	0.95
18. Ability to proceed with the project appropriately.		
19. Proceed with the project fairly.		
20. Proceed with the project with the same values as residents.		

* Reversed question

The survey also included questions on opinions about the final disposal, intergenerational subjective norm, social benefit, personal benefit, and the moral foundation, but these were not included in the present analysis. All of the questions were asked in Japanese. The questionnaire is available from the corresponding author upon request. Data of each participant are shown in S1 Table.

### Analysis

The responses to the conjoint questions were analyzed using the conditional logit model proposed by McFadden [28] to estimate the coefficients of a utility function. Several methods such as a the multinomial probit model are available for alleviating the limitations of the conditional logit model, which includes the assumption of independence of irrelevant alternatives (IIA assumption). However, the conditional logit model was considered sufficient in the present study because we focused on capturing the overview of public preference for multiple attributes of the final disposal.

As shown in Fig 1, we obtained responses on a scale of 1 to 6 for each pair of profiles, with responses 1 to 3 as case A choices, and responses 4 to 6 as case B choices. When individual *k* chooses profile *i*, the utility *U*_*ki*_ is as follows:

$$U_{ki}=V_{ki}+\varepsilon_{ki}=\beta_{ki}x_{ki}+\varepsilon_{ki}\;(1\leq i\leq I),$$

where *x*_ki_ is the attribute level of profile *i*, and *β*_ki_ is the utility parameter of individual *k*, a variable that varies randomly from individual to individual according to a probability distribution. *V*_ki_ is an observable (systematic) component of the utility and *ε*_ki_ is an unobservable disturbance term.

The probability that individual *k* chooses profile *i* as the most preferable, *P*_ki_, is calculated as follows:

$$P_{ki}=Pr[\max\left(U_{k1},\cdots ,U_{kI}\right)=U_{ki}]$$

When the disturbance term, *ε*_ki_, is independently and identically distributed according to the IIA assumption, *P*_ki_ is described as follows:

$$P_{ki}=exp(\beta_{ki}x_{ki})/\sum exp(\beta_{ki}x_{ki})$$

The *β*_ki_ is estimated by maximizing the value of the log-likelihood function. Each attribute level is not a continuous variable in the present study, and a dummy variable was used for each level. The observable component of the utility is modeled in this study as follows:

$$V=\beta_{1}x_{M}+\beta_{2}x_{P}+\beta_{3}x_{C}+\beta_{4}x_{D}+\beta_{5}x_{E}+\beta_{6}x_{F}+\beta_{7}x_{V}+\beta_{8}x_{S},$$

where *x*_M_ is a dummy variable that indicates siting within a municipality, *x*_P_ indicates siting within a prefecture, *x*_C_ indicates decision-making with offering comments, *x*_D_ indicates decision-making after discourse, *x*_E_ indicates siting in eight locations, *x*_F_ indicates siting in 46 locations, *x*_V_ indicates applying volume reduction, and *x*_S_ indicates applying super volume reduction. For each attribute, the following variables, namely located in a neighborhood, top-down decision-making, only one siting location in Japan, and no volume reduction treatment, were used as references. The remaining two attribute levels for each were variables that could be explained by parameters. The estimation of *β* was done using the function clogit() in the survival package of R [29].

To determine if there were groups of respondents for whom different attributes were more important, utility values were calculated by grouping respondents as described above, based on their basic knowledge about the final disposal, risk perception of the effects of radioactive materials on health, and trust in the Ministry of the Environment.

## Results

Table 4 shows the coefficient estimates of the observable component of the utility, *V*, obtained for the final disposal of the removed soil and the incinerated ash, respectively, by the conjoint analysis. The attributes for location, decision process, and number of sites had a statistically significant effect on the respondents’ preference (*p* < 0.05), while the volume and radioactivity had no effect. Respondents positively valued the distance of the disposal site from their residential area. For example, the coefficient estimates for siting the removed soil and incinerated ash within the municipality were 0.26 and 0.29 higher than for the neighborhood, while those for the removed soil and the incinerated ash were 0.56 and 0.59 higher for the prefecture than for the neighborhood. The coefficient estimates for a decision process where residents were able to offer comments were 0.41 and 0.39 for the removed soil and the incinerated ash, respectively. The coefficient estimates for a decision process where opinions were discussed were 0.52 for the removed soil and 0.45 for the incinerated ash. The coefficient estimates for having a total of eight disposal sites were 0.43 for the removed soil and 0.42 for the incinerated ash. The coefficient estimates for having one site in each of the 46 prefectures except Fukushima were 0.72 for the removed soil and 0.74 for the incinerated ash.

**Table 4:** Conjoint analysis results.

Attribute	Level	Removed soil		Incinerated ash	
		Coefficient	p-value	Coefficient	p-value
Location	Municipality	0.26	<0.01	0.29	<0.01
Ref = Neighborhood	Prefecture	0.56	<0.01	0.59	<0.01
Decision process	Comments	0.41	<0.01	0.39	<0.01
Ref = Top-down	Reflection of opinions	0.52	<0.01	0.45	<0.01
Number of sites	Eight	0.43	<0.01	0.42	<0.01
Ref = Only one	46	0.72	<0.01	0.74	<0.01
Volume and activity	Volume reduction	-0.04	0.23	0.01	0.75
Ref = No treatment	Super volume reduction	-0.04	0.20	-0.02	0.44
Likelihood ratio test		623.5		603.0	
p-value		< 0.00		< 0.00	

**Fig 2:**
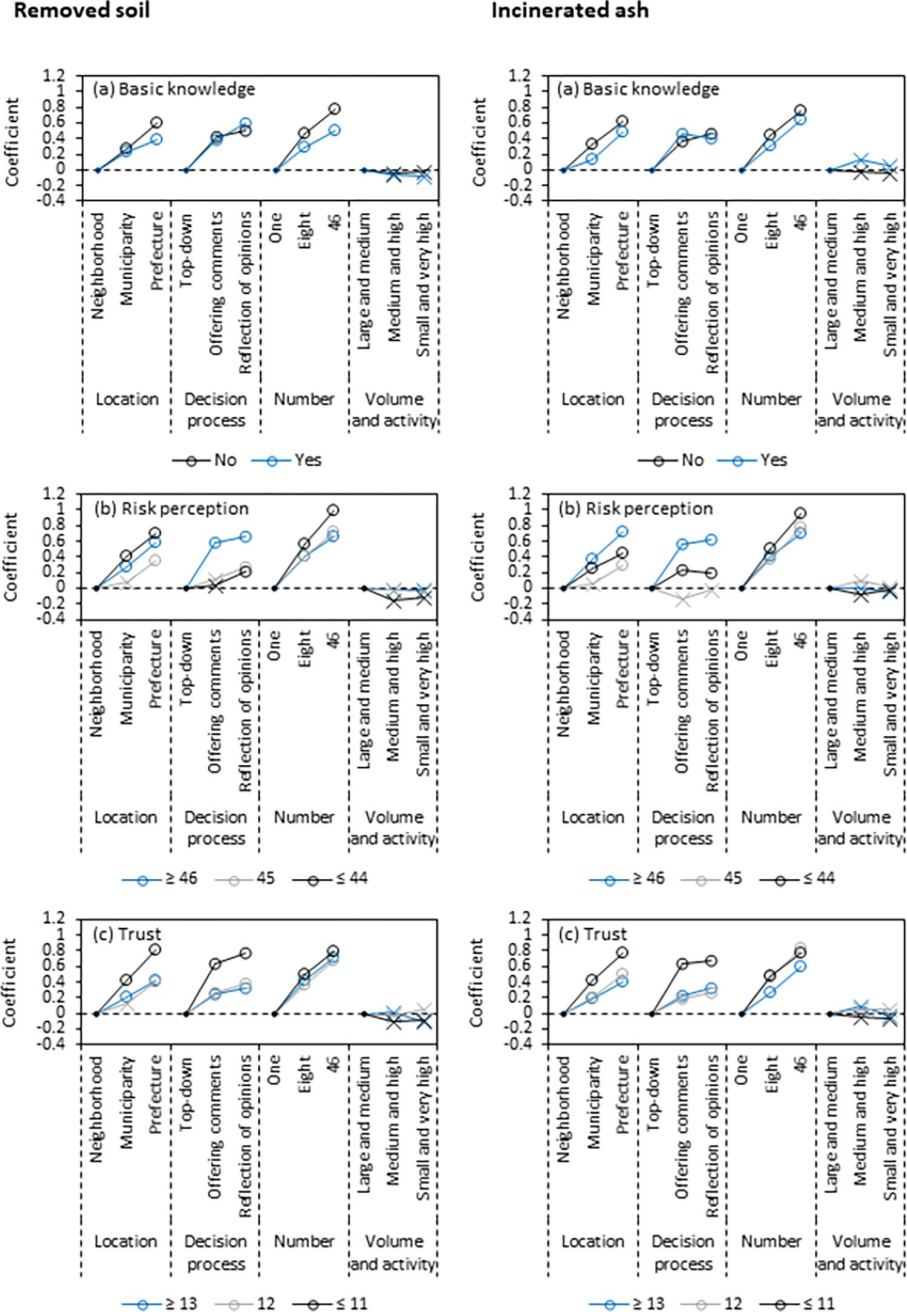
Coefficient estimates by response type for basic knowledge of final disposal, risk perception of health effects from radioactive materials, and trust in the Ministry of the Environment. The coefficients for neighborhood, top-down, one location, and no volume reduction (large and medium in volume and activity) were defined as references, and are shown by black circles in Fig 2. Open circles indicate statistical significance and cross marks indicate no statistical significance.

We examined the coefficient estimates by response type for the basic knowledge, risk perception, and trust, to determine if there were groups of respondents for whom different attributes were more important (Fig 2). The trend was small in the group with the basic knowledge for the removed soil, even within the trend of preferring disposal sites at multiple locations. The coefficient estimates for 8 and 46 locations without basic knowledge were 0.46 and 0.77, respectively, but were 0.29 and 0.50 with knowledge (Fig 2A).

The group with the high risk perception preferred the ‘offering comments’ and ‘reflection of opinions’ processes more than the top-down process. For the removed soil, the coefficient estimates for the low risk perception group were 0.03 and 0.21 for the ‘offering comments’ and ‘reflection of opinions,’ while those for the high risk perception group were 0.58 and 0.66, respectively. For the incinerated ash, the coefficient estimates for the low risk perception group for ‘offering comments’ and ‘reflection of opinions’ were 0.23 and 0.22, while those for the high risk perception group were 0.55 and 0.62, respectively (Fig 2B).

The group with less trust in the Ministry of the Environment preferred the ‘offering comments’ and ‘reflection of opinions’ processes. For the removed soil, the coefficient estimates for ‘offering comments’ and ‘reflection of opinions’ were 0.25 and 0.32 for the high trust group, respectively, and 0.64 and 0.78 for the low trust group. For the incinerated ash, the coefficient estimates for ‘offering comments’ and ‘reflection of opinions’ were 0.22 and 0.33 for the high trust group, respectively, and 0.64 and 0.67 for the low trust group (Fig 2C).

## Discussion

### Location of final disposal sites

Many previous studies have shown that the acceptance decreases as the distance between the disposal site and residential area decreases (e.g., [6–9]). Previous studies involving several waste disposal sites have observed that the relationship between the distance and acceptance (or resistance) is not always linear, and that an increase in the distance has little effect on the acceptance or resistance beyond several to tens of kilometers. For example, the distance was 30 km in the study of Lober & Green [6], and 5 km in studies by Akiyama et al. [9] and Sasao [23]. However, unlike previous studies, the preference in this study did not reach a plateau as the distance increased. The differences between the coefficient estimates of the municipality and the neighborhood and the municipality and the prefecture were similar, perhaps because the question was about abstract distances, not concrete distances. The relationships between the distance of the disposal site from the respondent and the preference are intuitive and easy to understand. We can compare these with the preference of other attributes to help us understand the results of the other attributes.

### Decision process

The preference was higher when residents could influence decision-making by offering comments and reflecting on opinions than for the top-down decision process. The preference of the prefectures was comparable, even those for the most distant prefecture, indicating the high importance of procedural fairness, which is consistent with the results of many previous studies [13–15]. When the siting of a waste facility is seen as a top-down or imposed decision [30], such an approach often fails [14].

Respondent groups with higher risk perception and lower trust in the Ministry of Environment showed a particular preference for stakeholder-involved decision making. Previous studies reported that high risk perception and low trust in government institutions hinder acceptance [10, 11]. The present result is consistent with what these previous studies suggest, and indicates that procedural fairness is particularly likely to be associated with acceptance, especially for those with high-risk perception and low trust.

### Total number of final disposal sites

As the total number of final disposal sites increased, the preference also increased. When there were eight locations, one in each of the eight regional divisions, the preference was equal to that for procedural fairness. The preference was even high, and was the highest in this study, when there were 46 locations, one in all the prefectures except Fukushima. This suggests that many respondents felt it was unfair to locate a final disposal site only close to their residential area. Yokoyama et al. [19] showed that acceptance was encouraged by pursuing distributive fairness if recycled decontaminated soil was used in multiple places, which is consistent with the results of this study. In addition, our results showed that the relative importance of distributional fairness through sharing the actual burden was greater than that of procedural fairness. This high importance may reflect the characteristics of the final disposal. As shown in Table 1, these characteristics may have increased the respondents’ awareness of ‘Why only us?’, and consequently, the preference was high for a level of burden-sharing across Japan.

Although the relative importance of distributive fairness through multiple sites tended to be higher, the group without basic knowledge of the final disposal showed a greater preference for multiple sites for the removed soil, while the group with the knowledge showed a higher preference for one site location. It is possible that the respondents with the knowledge did not have strong preferences for multiple site locations because they found it difficult to imagine how it could be realized. Alternatively, it is possible that respondents with knowledge after 25 years included those who were completely opposed to the final disposal plan, and so the increase in the number of sites had little effect on their preferences.

### Volume and radioactivity

The results for the removed soil and the incinerated ash were very similar, even though the respondents were different and the volume and radioactivity with the preliminary explanations were also very different (S1 Fig). This implies that the volume and radioactivity did not affect the respondents’ preferences, and the coefficient estimates for this attribute were not statistically significant.

The result may reflect the respondents’ lack of understanding and responsibility about the final disposal. In a web-based survey conducted by the Ministry of the Environment in 2020, approximately 80% of 3500 respondents outside Fukushima Prefecture said they knew nothing about the potential for final disposal outside of the prefecture [31]. It is easy to imagine that a lack of understanding and responsibility would lead to a lack of preference for the detailed and specific conditions of the disposal site. Otherwise, the risk related to the radioactivity and the size of the disposal site based on the volume may have been difficult to visualize concretely for most respondents because of the lack of technical information, meaning that this attribute was somewhat less important than the other three attributes.

### Implications

Our results showed that the public did not like having a final disposal site near their residential area, which is understandable, but they also showed a negative reaction for sites only close to their area. Based on the concept of burden sharing, a plan for multiple sites rather than one site would be fair. However, it may be impractical to have multiple final disposal sites throughout Japan because the efficiency of managing multiple sites would be significantly lower than for one site. A group of respondents with less basic knowledge tended to prefer multiple locations, which suggests that the importance could change in the future if the public had a better understanding of, and sense of responsibility for, the process. In addition, regardless of the number of disposal sites, it is impossible to completely correct any unfairness in the distribution because there are people living both close to and far from each disposal site. Accordingly, it is necessary to pursue consensus along with a fair procedure, in line with the public’s preference, as suggested in previous studies [19, 32]. It may be possible to reduce the difficulties in acceptance because of the perception of high risk and low trust in the government by ensuring that the decision process follows a fair procedure.

It is noteworthy that the volume and radioactivity do not always affect the public preference or acceptance. This attribute has a significant impact on the local conditions in which the disposal site is to be built. The relative importance of this issue may increase as society becomes more aware of the final disposal, the candidate sites are narrowed down, and local residents gain a sense of ownership. Therefore, the Japanese government should be cautious about making hasty decisions concerning volume reduction policies, as volume reduction is an irreversible process.

Many researchers have examined how fairness impacts the public acceptance of NIMBY facilities, but few have examined how to directly mitigate inequity through having multiple sites. To the best of our knowledge, this is the first time that the preference for different types of fairness have been compared, with the novel finding that the preference for direct inequity mitigation was very high, and higher than that for procedural fairness. While the public easily accepts the idea of ‘sharing the burden’ for waste disposal, it is only practical in some cases, such as when there are no geographical limitations, the candidate area is large, and the need for final disposal results from a disaster.

### Limitations

We used a web-based questionnaire survey and received responses from 4000 people. We may have biased the respondents by doing this survey on the internet. Therefore, the result should be validated using other methods, such as a mail survey. For the volume and radioactivity attribute, the respondents may not have understood the implications of the difference in the levels. This preference should be examined through additional surveys using interviews rather than questionnaires. Furthermore, if the lack of preference for the volume and radioactivity attribute was influenced by the respondents’ lack of understanding and responsibility for the final disposal, the importance should be surveyed periodically. The conditional logit model used in the present analysis provides an analytical solution, but has the disadvantage of the assumption of independence of irrelevant alternatives (IIA assumption). The future validation survey addressed above should consider more appropriate models that relax the IIA assumption. Despite the limitations mentioned above, the results for the removed soil and the incinerated ash were very similar, which means that the public’s preferences for the distance and the fairness attributes were robust.

## Conclusions

In the present study, the relative importance of a range of factors, namely the distance between the disposal site and the residential area, two types of fairness (procedural fairness and distributive fairness), and the volume and radioactivity, were examined by choice-based conjoint analysis, with a timing of 25 years set for the completion of the final disposal of contaminated soil and wastes generated as a result of the Fukushima Daiichi nuclear power station accident in 2011 outside of the prefecture. The results showed that fairness in the implementation of this final disposal plan was particularly important. Of the types of fairness, the preference for direct inequity mitigation by using multiple locations was higher than that for procedural fairness. In addition, there was no preference for the volume and radioactivity of the substances. At the time of our survey, the degree of public recognition of the completion of the final disposal outside of Fukushima Prefecture by 2045 was not very high. We would like to continue the survey because the importance of these factors may change as public understanding increases.

## Supporting information

S1 Table All responses for removed soil and incinerated ash. (XLSX)

S1 Fig Information about what the respondent watched before answering. Duration for which control was required, storage method, and air dose in the vicinity when volume reduction was applied to the removed soil and incinerated ash.
(TIF)
