# Supplementary figures and images for: Important factors for public acceptance of the final disposal of contaminated soil and wastes resulting from the Fukushima Daiichi nuclear power station accident
Source: PLoS One. 2022 Jun 22;17(6):e0269702. doi: 10.1371/journal.pone.0269702 (PMC9216558; doi:10.1371/journal.pone.0269702)

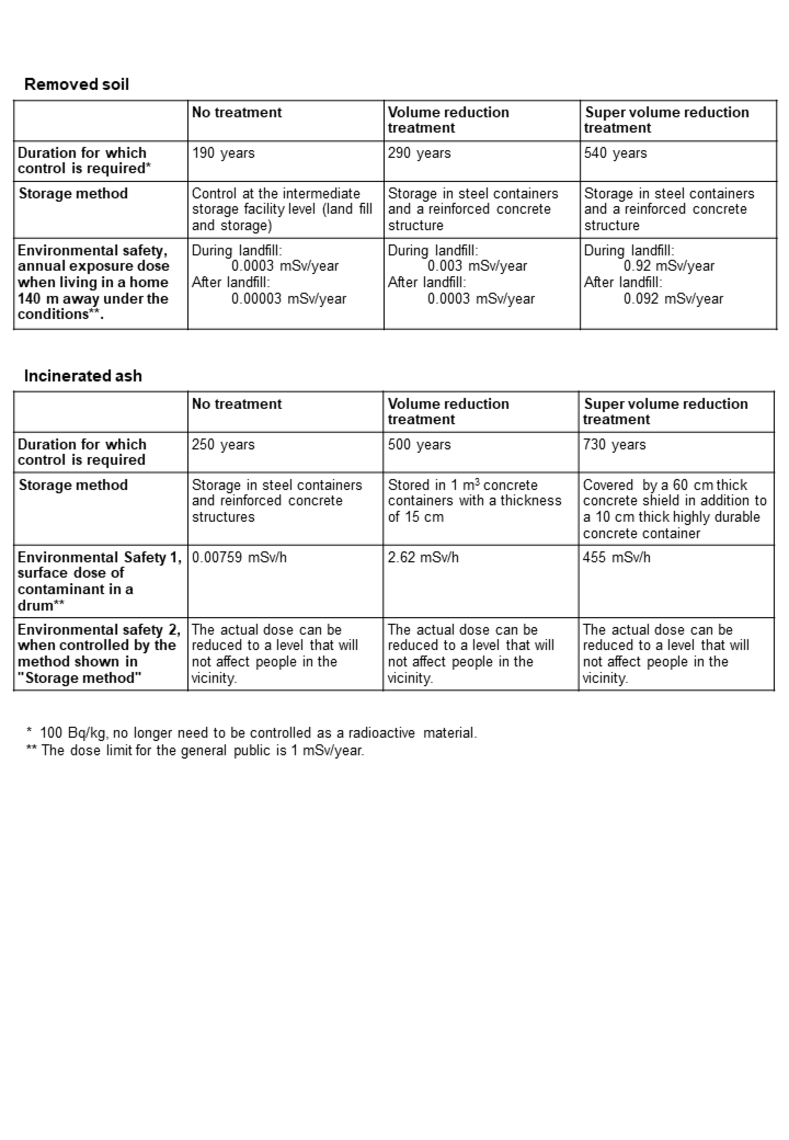

Supplement: S1 Fig — Duration for which control was required, storage method, and air dose in the vicinity when volume reduction was applied to the removed soil and incinerated ash. (TIF) [file pone.0269702.s002.tif]
